# Mid-Term Outcomes of a Short Modular Neck-Preserving Cementless Hip Stem: A Retrospective Study With a 6-Year Minimum Follow-Up

**DOI:** 10.1016/j.artd.2024.101387

**Published:** 2024-04-27

**Authors:** Michele Carnovale, Daniele De Meo, Giovanni Guarascio, Paolo Martini, Gianluca Cera, Pietro Persiani, Vittorio Candela, Stefano Gumina, Ciro Villani

**Affiliations:** a Emergency Department, Policlinico Umberto I University Hospital, Rome, Italy; b Department of Anatomical, Histological, Forensic Medicine and Orthopaedics Sciences, Sapienza University of Rome, Rome, Italy; c Department of General Surgery, Plastic Surgery and Orthopaedics, Policlinico Umberto I University Hospital, Rome, Italy; d Istituto Clinico Ortopedico Traumatologico (ICOT), Latina, Italy

**Keywords:** Primary, Total hip arthroplasty, Modularity, Short stem, Neck-preserving

## Abstract

**Background:**

The neck-preserving cementless short stem represents a valid therapeutic option for total hip replacement in high-functional-demand patients, but few studies are available about the use of modularity in the last-generation short stem. The aim of the study was to evaluate the mid-term survival of a specific implant design that combines partial collum short hip stem with neck modularity; assessing the functional status was the second endpoint.

**Methods:**

A retrospective single-center cohort study was conducted on 75 patients aged 35 to 80 years, with a minimum 6-year follow-up. Patients with neurological/rheumatic pathologies and previous hip surgeries were excluded. All the patients underwent total hip replacement with a short modular neck-preserving cementless hip stem. Clinical outcomes, complications, revisions, and the Western Ontario and McMaster Universities Osteoarthritis Index, Harris hip score, and Short Form 12-Item Health Survey (SF-12) questionnaires were evaluated. The results were compared with healthy population’s data extracted from the literature, stratified by age.

**Results:**

The Kaplan-Meier analysis revealed a 10-year implant survival rate of 96.7%, coupled with a revision rate of 1.3%. Results showed a Harris hip score and physical SF-12 significantly lower and a mental SF-12 higher when compared to healthy population. No statistically significant differences emerged when comparing groups based on neck modularity.

**Conclusions:**

The short modular neck-preserving cementless hip stem emerged as a reasonable choice for patients with elevated functional demands, ensuring good clinical outcomes while preserving bone integrity. The use of a modular neck in short stems didn’t show any mechanical problems in the mid-term.

## Introduction

The hip arthroplasty procedure in the last century has revolutionized the patient’s treatment of osteoarthritis [1].

Over the years, hip replacement surgery has primarily targeted patients over the age of 70 with generally low functional demands [2]. However, scientific progress has led to an increase in life expectancy and, simultaneously, higher functional patients’ demands. This shift has gradually diminished the significance of age as a parameter in the selection and indication for prosthetic replacement [3]. Indeed, age has been overshadowed by the evaluation of the patient's activity level, which is crucial in determining the appropriate implant. As early as 2015, Torre et al. highlighted a global increase in the number of hip prostheses implanted in Italy, even in younger patients, both those under 65 years old and even under 55 years old, representing a 141% increase on an epidemiological analysis spanning from 2001 to 2013 [4].

**Figure 1:**
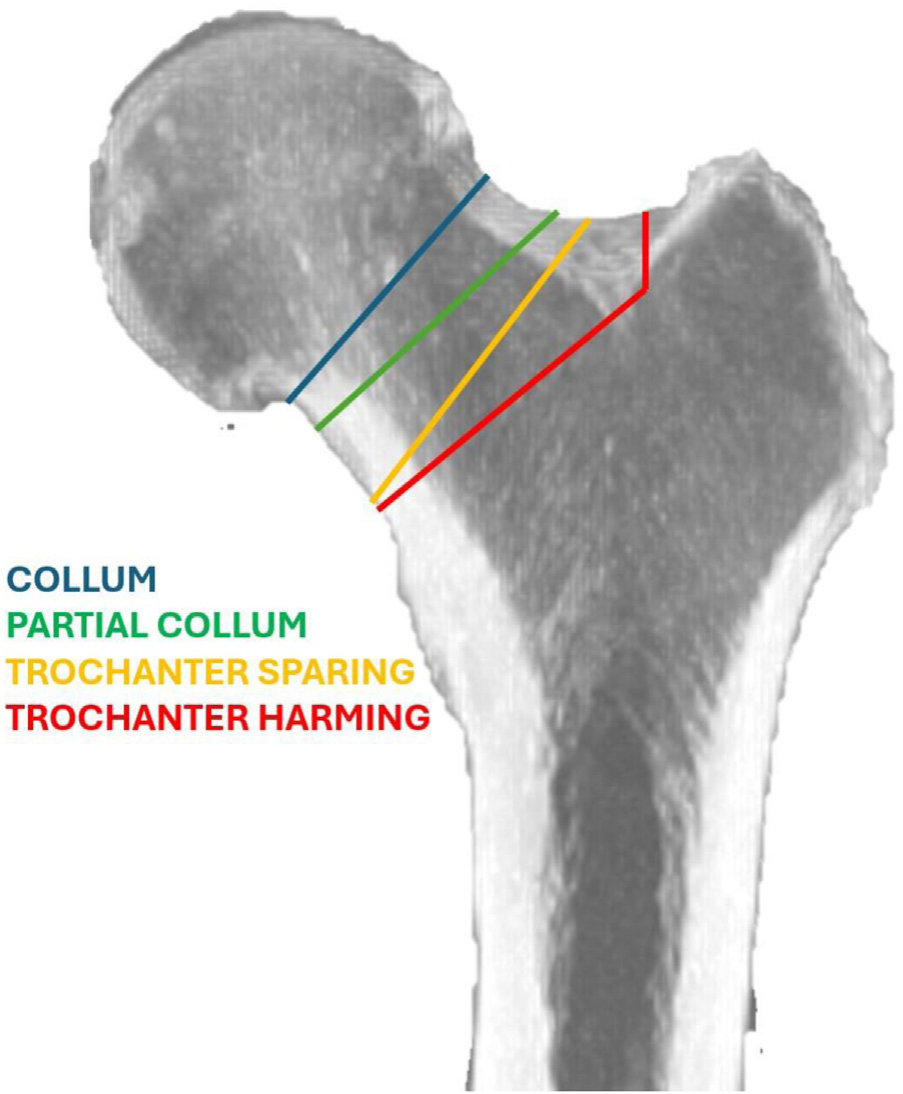
Falez et al. classification of femoral implants based upon the level of neck resection [7].

From an engineering perspective, hip arthroplasty has witnessed significant innovations over the years. Tribological studies have led to the experimentation of new materials such as high-performance ceramic and cross-linked polyethylene [5]. Software simulations and computer-aided design systems have led to new designs of femoral stems that better replicate biomechanics and femoral anatomy, maximizing the preservation of the patient's bone structure in joint replacement procedures. Currently, hip stems can be distinguished between standard femoral stems, short stems, and resurfacing hip replacements [6]. Within the category of short stems, we can further differentiate implants based on their level of femoral neck resection, according to the classification proposed by Falez et al. This classification takes femoral osteotomy into account and distinguishes 4 types of stems: collum, partial collum, trochanteric sparing, and trochanteric harming (Fig. 1) [7].

Orthopaedic surgeons are faced with a challenge in treating patients with hip arthroplasty, often involving a cohort of young and/or active patients from a sports perspective with high functional demands. In this context, the choice of the most suitable implant becomes crucial.

Literature data suggests that standard stems have favorable outcomes but sacrifice more femoral bone, and patients may experience thigh pain in the long term [8,9]. On the other hand, the resurfacing prosthesis maximizes bone stock preservation, but its indication is narrow and its use is not widespread [10]. Currently, according to data from the Italian Joint Registry and Australian Registry, resurfacing prostheses account for only 0.4% and 1% of elective implants, respectively [11,12]. Additionally, Amstutz et al. recommend this procedure only for male patients under 50 years old with a femoral head diameter greater than 48 mm [13].

In recent years, the concept of short-stem prostheses with neck preservation has gained increasing interest as a therapeutic option. However, the literature data on this topic are quantitatively and qualitatively inferior compared to other types of implants, particularly concerning implants designed and manufactured with the latest generation of design, technology, and materials. Collum and partial collum stem have the advantage of a bone sparing technique, allowing a more feasible revision. In this setting, there is a paucity of data regarding the use of modular neck stems. Restoration of the biomechanical anatomy is one of the main goals of total hip arthroplasty (THA), which can be challenging. To solve this issue, modular neck stems were used with initial failures—mostly related to the combined use of different types of material for the stem and the neck—that new technologies seem to have solved [14]. The use of a short stem with a modular neck, theoretically, combines all the advantages of these 2 techniques to apply it in a setting in which a high-performance implant is needed the most, ie, young active patients. There are few stem designs that allow this combination of features within next-generation coating techniques.

Therefore, the aim of our study was to verify whether the use of a partial collum short hip stem with modular neck can be a safe and effective option for active subjects with good bone quality in the medium term. We first studied as our primary outcome the medium-term survival of the implant and as our secondary outcome the functionality according to the different types of modular neck version, length, and inclination.

## Material and methods

### Patients and methods

A retrospective single-center study has been conducted in the Orthopedic Department of Sapienza University, including all the patients who underwent surgery for primary and/or secondary hip arthritis and avascular necrosis of the femoral head from June 1, 2012, to September 30, 2017. Inclusion criteria were: application of a partial collum short hip stem with modular neck in the setting of a THA; patients aged between 35 and 80 years; same surgical approach (posterolateral approach to the hip) and equipe; minimum follow-up of 6 years (72 months).

Exclusion criteria were: patients with previous hip surgeries for any reason, revisions THA, previous infections, cemented femoral stem; patients with chronic inflammatory diseases (ie, rheumatoid arthritis); patients with degenerative neurological diseases or those affected by localized cancer or bone metastasis; patients affected by end-stage cancer (prognosis <6 months).

The implant used was Parva stem (AdlerOrtho, Cormano, Italy), ie, a short stem with preservation of the neck characterized by a coating obtained through powder technology and electron beam melting, a method that allows for a highly porous titanium alloy coating with low corrosion rates and high resistance to delamination. [15,16]. The Modula neck, entirely made of titanium alloy, allows for independent modification of the 3 geometric parameters of the joint: offset, length, and version.

**Figure 2:**
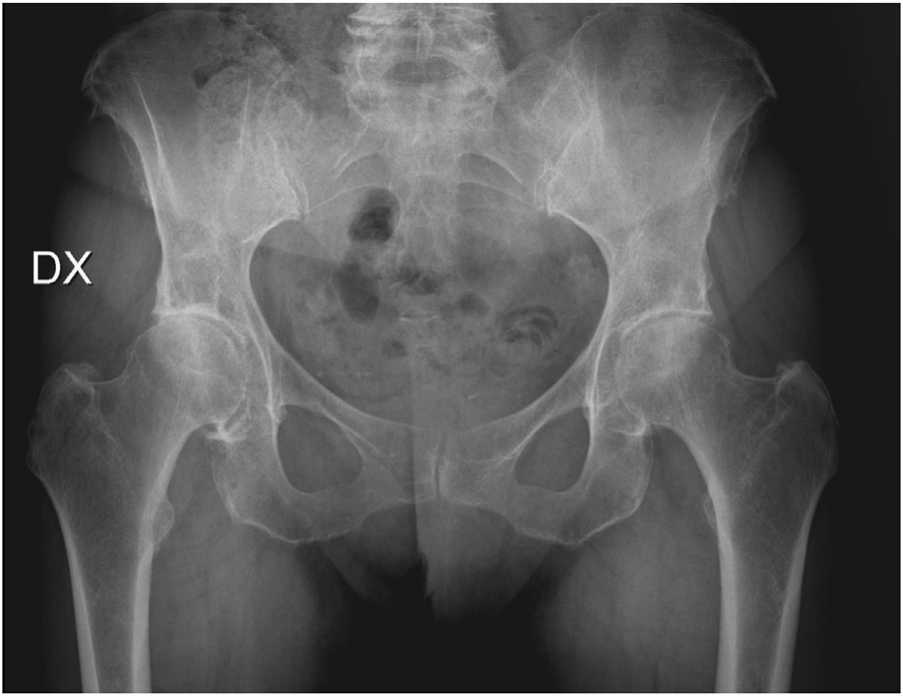
Preoperative x-ray of bilateral hip osteoarthritis in a 67-year-old woman.

**Figure 3:**
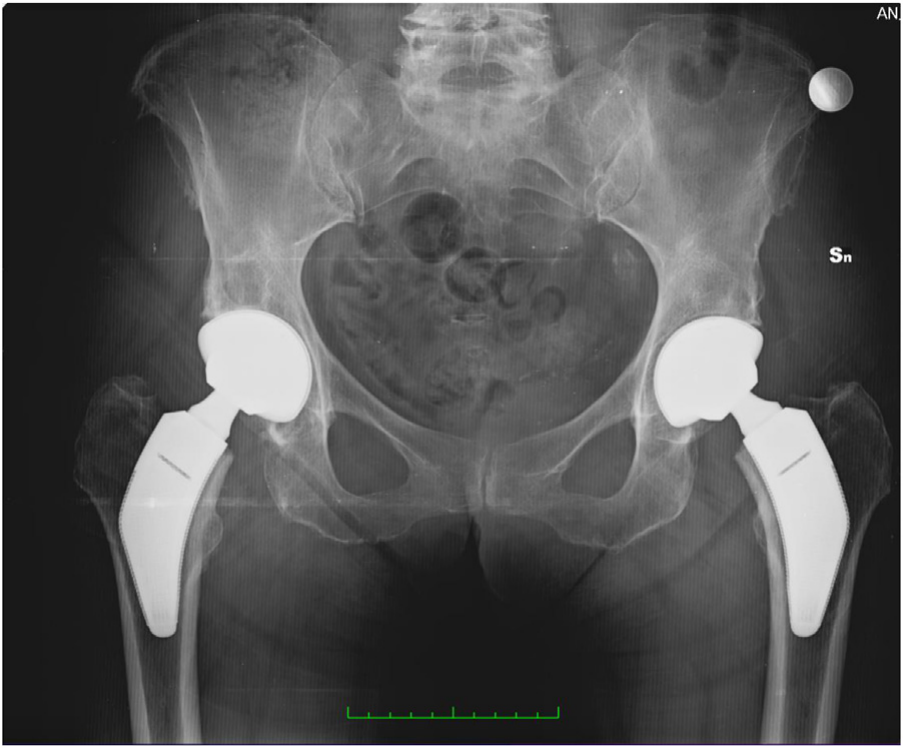
Six-year follow-up x-ray of a bilateral total hip arthroplasty.

Among the nearly 500 THA performed by the same surgeon over this study period, only patients that fit the inclusion criteria were enrolled. Seventy-five patients satisfied the aforementioned inclusion and exclusion criteria and were enrolled in the study. All patients were operated on by the same surgeon, who also had the most years of experience (C.V.) (Figure 2, Figure 3). The implant’s choice was made by the operating surgeon and confirmed by a preoperative meeting in which the planning was discussed and approved case-by-case. Parameters usually considered were age, body mass index (BMI) (patients with a BMI >40 were usually sent to bariatric surgery before proposing surgery with any type of stem), Dorr classification-type C were usually excluded, overall bone quality, and patient activity. An antibiotic dose (Cefazolin 2g or Vancomycin 1g in case of allergy) was administered according to hospital guidelines. All the patients underwent spinal anesthesia and pericapsular nerve group blocks. Standard postoperative rehabilitation protocol included full weight-bearing and assisted ambulation, along with isometric muscle strengthening exercises, starting from postoperative day one. The standard rehabilitation protocol lasted approximately 4 weeks postsurgery in the setting of fast-track surgery [17].

Demographic data of the sample, the type of implant used, complications, and inpatient outcomes were obtained from the hospital digital record archive. All patients who underwent THA procedures were routinely examined at the outpatient clinics at periodic intervals (1-3-6 months and annually). For the purpose of this study, during the last follow-up visit, a clinical examination and collection of internationally validated questionnaires were performed. The stem survival (stem revision for any cause, excluding infection) and the implant survival (implant revision for any cause, including infection) were recorded to evaluate the primary outcomes. To assess the secondary outcomes, the following questionnaires were performed: visual analog scale [18], the Western Ontario and McMaster Universities Osteoarthritis Index (WOMAC) [19], the Harris hip score (HHS) [20], the Short Form 12-Item Health Survey (SF-12) [21] within the2 physical component summary (PCS) and mental component summary (MCS) [22,23]. A radiographic evaluation was performed, and any complication was recorded. Once the respective values were obtained, the results, expressed as means and standard deviations, were compared with literature data from a healthy populations, stratified by age [[24], [25], [26]].

The local ethics committee exempted the study from approval, which is not necessary according to the law of our country. Informed consent was obtained from all the individual participants included in the study.

The study was conducted with informed consent obtained from all patients and in accordance with the principles of the Declaration of Helsinki and Good Clinical Practice guidelines (E6: Good Clinical Practice: Consolidated Guideline [CPMP/ICH/135/95]).

### Statistical analysis

Statistical analysis was conducted using the R 4.2.2 software (R Foundation for Statistical Computing, Vienna, Austria). All obtained values are expressed as mean, standard deviation, and/or 95% confidence interval.

The level of significance for observed differences being due to chance is 0.05 (α-value). The sample size was calculated using the G∗Power 3.1.9.6 software (Heinrich-Heine-University Dusseldorf, Germany). Regarding the secondary outcome, we conducted an “a priori” analysis, resulting in the need for 57 patients considering an alpha value of 0.05, a beta value of 0.95 (study power of 95%), and a standard effect size of 0.5. The D’Agostino-Pearson test will verify the normal distribution of variables. Parametric tests will be used when this condition is met, and nonparametric tests will be used when this condition is not met. Regarding the primary outcome, the Kaplan-Meier analysis will estimate the implant survival percentage.

## Results

The total sample consists of 75 patients, 43 men (57.3%) and 32 women (42.7%) with a mean age of 58.87 ± 11.02. Among them, 63 patients (84%) underwent surgery for primary hip arthritis, 10 patients (13.4%) for avascular necrosis of the femoral head, one patient due to Perthes disease (1.3%), and one patient due to Paget's disease (1.3%). All patients received a ceramic-ceramic head-inlay coupling. Short heads were implanted in 37 patients (49.3%), medium heads in 32 patients (42.7%), and long heads in 6 patients (8%). Neutral necks were used in 57 patients (76%), while 18 patients received an anteverted neck (24%). Retroverted necks were not used. Necks with variation in inclination angle (varus-valgus) were used in 32 patients (42.6%). A medium neck was implanted in 19 patients (25.3%), and a short neck was implanted in 56 patients (74.7%). Long necks were not used.

**Figure 4:**
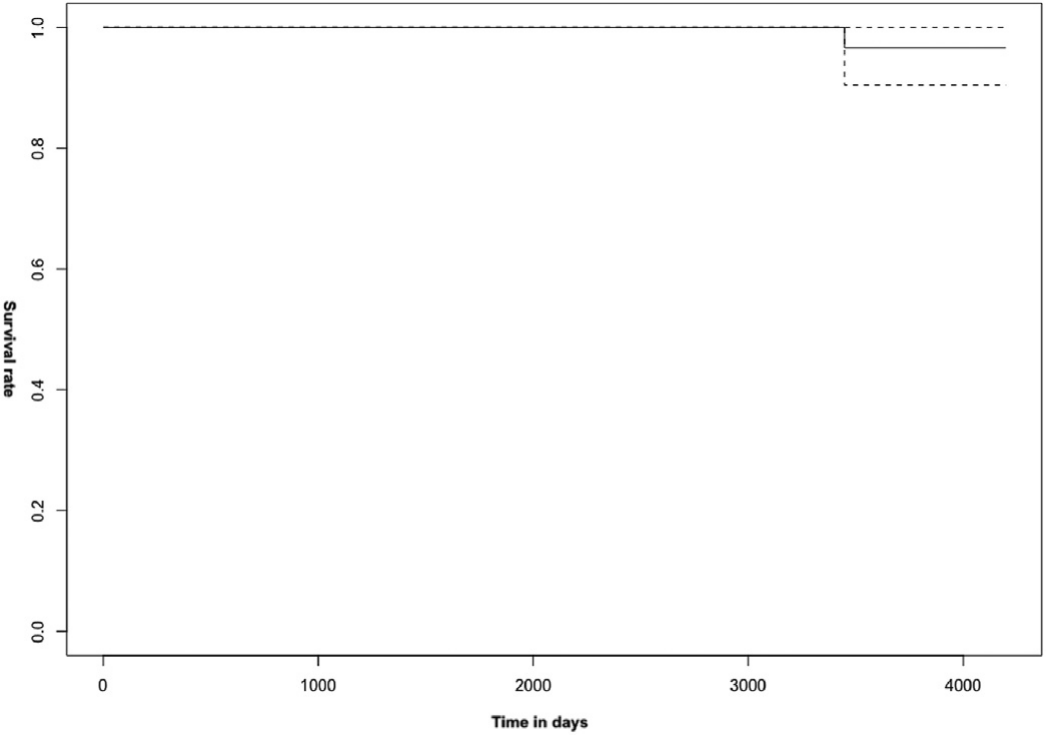
Kaplan-Meier survival curve of the implant.

Concerning the primary outcome, implant survival recorded through Kaplan-Meier analysis at 10 years is 96.7%, with a revision rate for any cause of 1.3% (Fig. 4).

No cases of prosthetic implant infection or dislocation were recorded. A B1-type periprosthetic fracture, according to the Vancouver classification, occurred in a female patient due to high-energy trauma and was subsequently treated with plate, cerclage, and screw-open reduction internal fixation (Table 1).

**Table 1:** Results related to the examined sample.

	N (%)
Total sample	75
Men	43 (57.3%)
Women	32 (42.7%)
Mean age (standard deviation)	58.87 (11.02)
Surgery reason	
Primary hip arthritis	63 (84%)
Avascular necrosis	10 (13.4%)
Perthes disease	1 (1.3%)
Paget disease	1 (1.3%)
Head type	
Short	37 (49.3%)
Medium	32 (42.7%)
Long	6 (8%)
Neck version	
Neutral	57 (76%)
Anteverted	18 (24%)
Neck inclination·	
Neutral	43 (57.4%)
Varus/valgus	32 (42.6%)
Neck length	
Short	56 (74.7%)
Medium	19 (25.3%)
Long	0
Complication	1 (1.3%)
Survival rate at 10 years	96.7%

Regarding secondary outcomes, clinical evaluation and patient-questionnaire results were compared with the one retrieved from the general healthy population: data showed a visual analog scale score of 3.73 out of 100 (standard deviation 4.88), the WOMAC score of 5.92 (confidence interval [CI] 5.04-6.79, *P*-value .17), the HHS of 93.65 (CI 92.56-94.73, *P*-value < .01). The obtained SF-12 questionnaire result is 49.88 (CI 48.51-51.24, *P*-value < .01) for PCS and 55.89 (CI 54.43-57.35, *P*-value < .01) for MCS (Table 2).

**Table 2:** Clinically observed outcomes in the total sample compared with literature data related to the healthy population.

Clinical score	Sample	Healthy population	*P*-value
VAS	3.73	<5	N.A.
WOMAC	5.92	5.82	.17
Harris hip score	93.65	95.64	**<.01**
SF-12			
PCS	49.88	50.3	**<.01**
MCS	55.89	51.3	**<.01**

N.A., not applicable; VAS, visual analog scale.

*P*-value <.01 are highlighted in bold.

**Figure S1:**
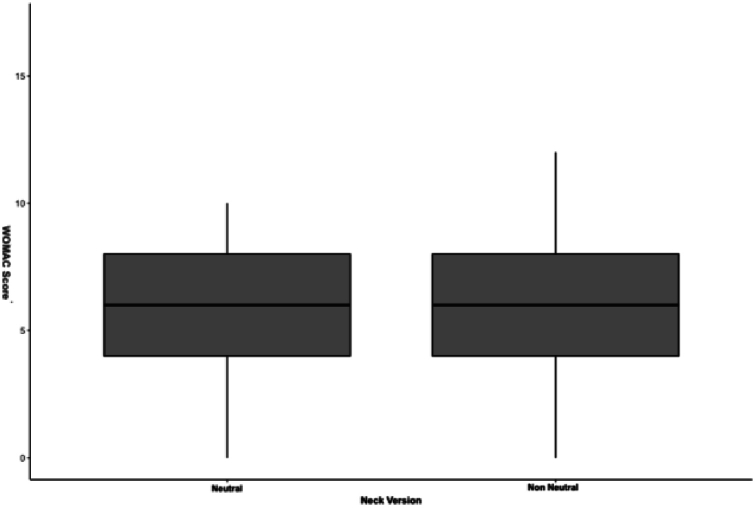
Box plot distribution of WOMAC score values in the “neutral neck” and “anteverted neck” groups, *P*-value = .89.

**Figure S2:**
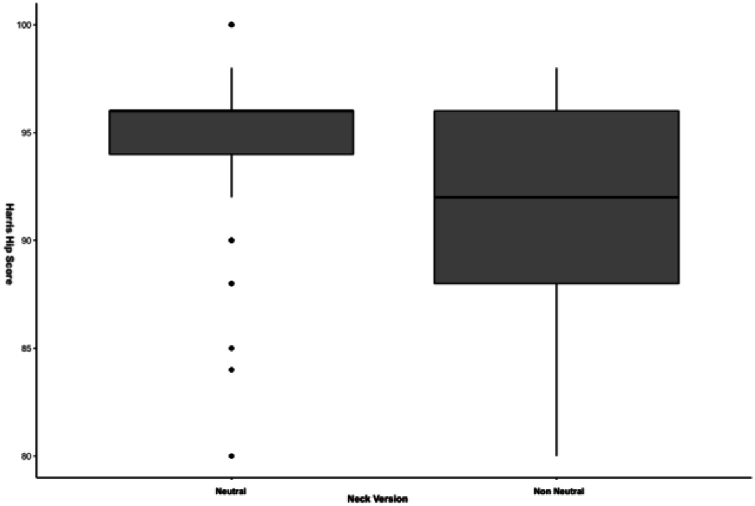
Box plot distribution of Harris hip score values in the “neutral neck” and “anteverted neck” groups, *P*-value = .06.

**Figure S3:**
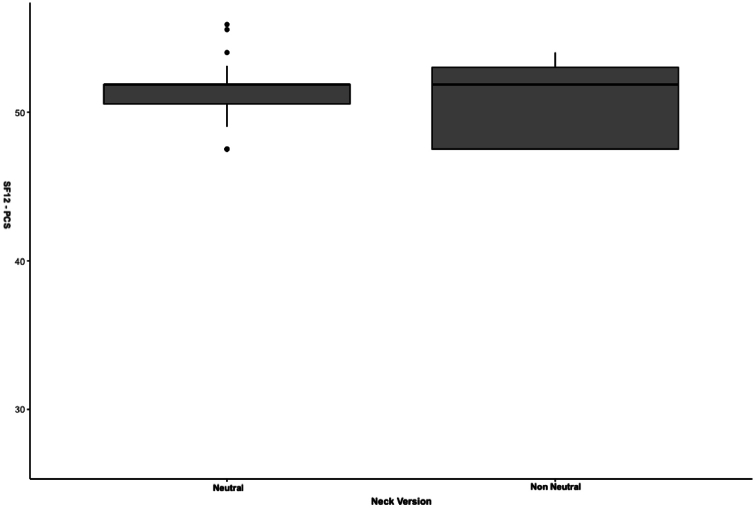
Box plot distribution of SF-12 PCS values in the “neutral neck” and “anteverted neck” groups, *P*-value = .91.

**Figure S4:**
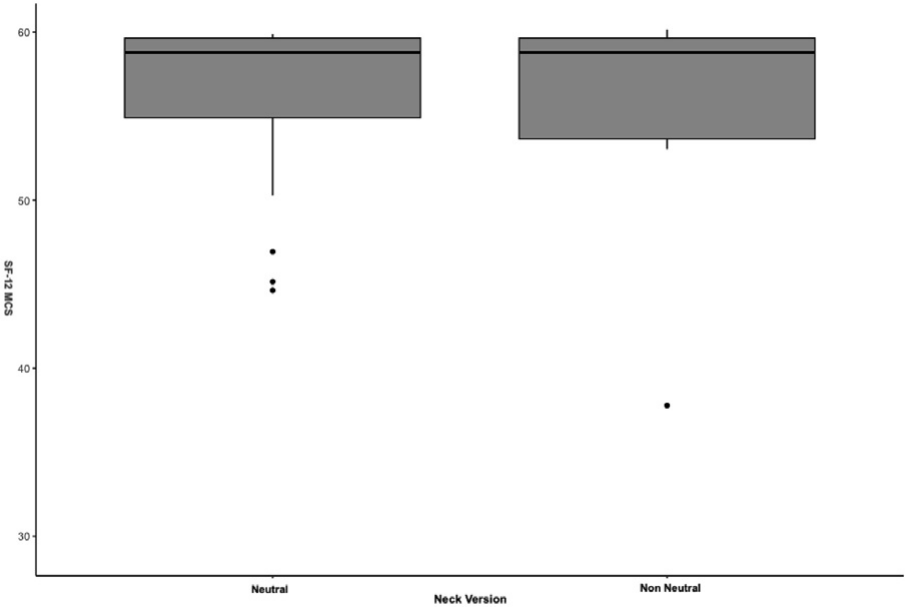
Box plot distribution of SF-12 MCS values in the “neutral neck” and “anteverted neck” groups, *P*-value = .657.

The following clinical outcomes were obtained by splitting the sample into subgroups with anteverted and neutral necks: WOMAC score of 5.94 and 5.82 in the neutral and anteverted neck groups, respectively (*P*-value = .895) (Fig. S1), HHS of 94.35 for neutral neck and 91.41 for anteverted neck (*P*-value = .06) (Fig. S2), SF-12 PCS of 50.51 for neutral neck and 47.85 for anteverted neck (*P*-value = .91) (Fig. S3), and 56.29 and 54.63 for SF-12 MCS in the neutral and anteverted neck groups, respectively (*P*-value = .657) (Fig. S4) (Table 3).

**Table 3:** Clinical outcomes in the subgroups obtained with a neutral neck and an anteverted neck.

	Neutral neck	Anteverted neck	*P*-value
Harris hip score	94.35	91.41	.06
WOMAC score	5.94	5.82	.895
SF-12			
PCS	50.51	47.85	.91
MCS	56.29	54.63	.657

**Figure S5:**
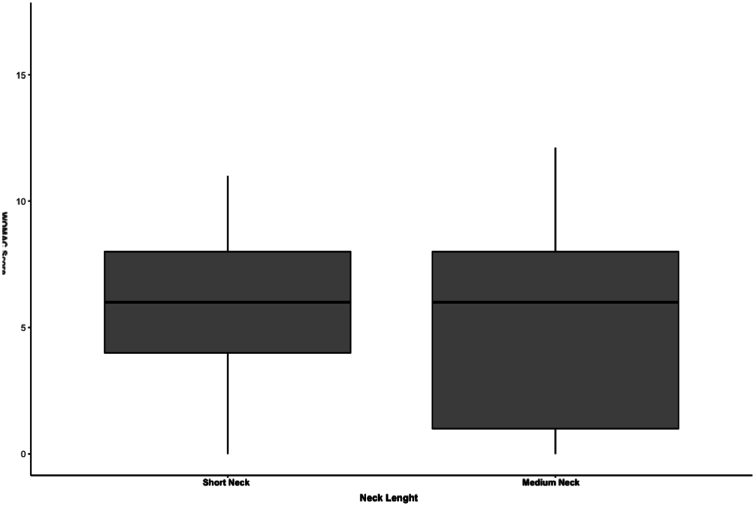
Box plot distribution of WOMAC score values in the “short neck” and “medium neck” groups, *P*-value = .95.

**Figure S6:**
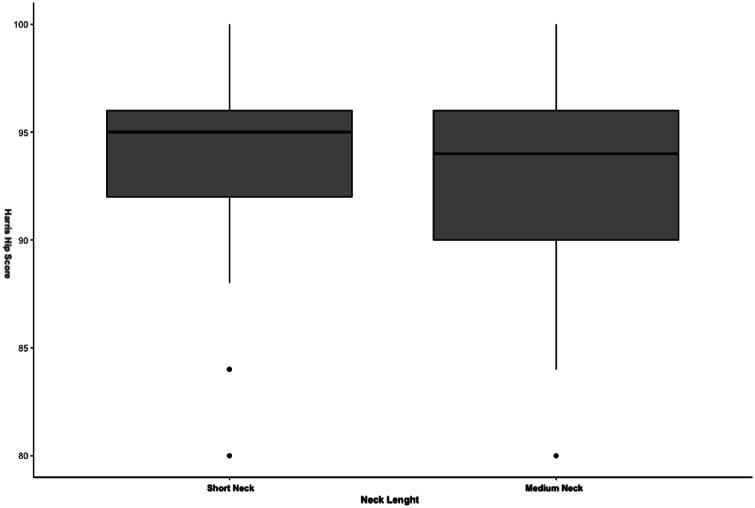
Box plot distribution of Harris hip score values in the “short neck” and “medium neck” groups, *P*-value = .77.

**Figure S7:**
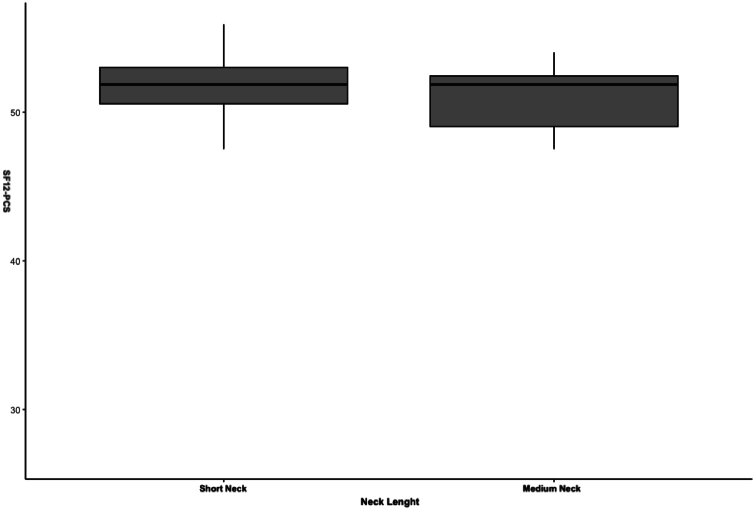
Box plot distribution of SF-12 PCS values in the “short neck” and “medium neck” groups, *P*-value = .68.

**Figure S8:**
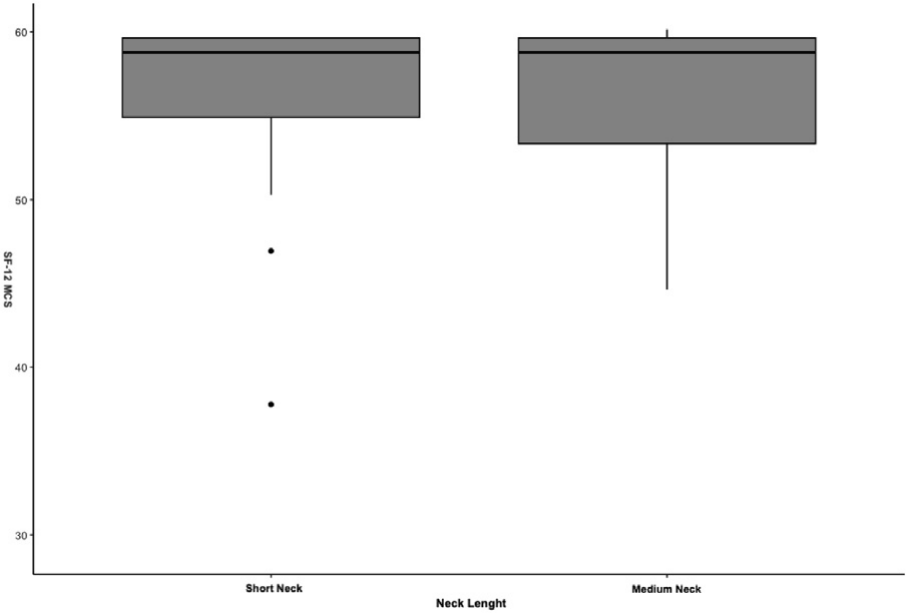
Box plot distribution of SF-12 MCS values in the “short neck” and “medium neck” groups, *P*-value = .85.

Subdividing the sample based on neck length yielded the following results: WOMAC score of 5.81 for short neck and 6.21 for medium neck (*P*-value = .95) (Fig. S5), HHS of 93.88 for short neck and 93 for medium neck (*P*-value = .77) (Fig. S6), SF-12 PCS of 50.43 for short neck and 48.37 for medium neck (*P*-value = .68) (Fig. S7), and 56.34 and 54.68 for SF-12 MCS in the short and medium neck groups, respectively (*P*-value = .85) (Fig. S8) (Table 4).

**Table 4:** Clinical outcomes in the subgroups obtained with short and medium necks.

	Short neck	Medium neck	*P*-value
Harris hip score	93.88	93	.77
WOMAC score	5.81	6.21	.95
SF-12			
PCS	50.43	48.37	.68
MCS	56.34	54.68	.85

**Figure S9:**
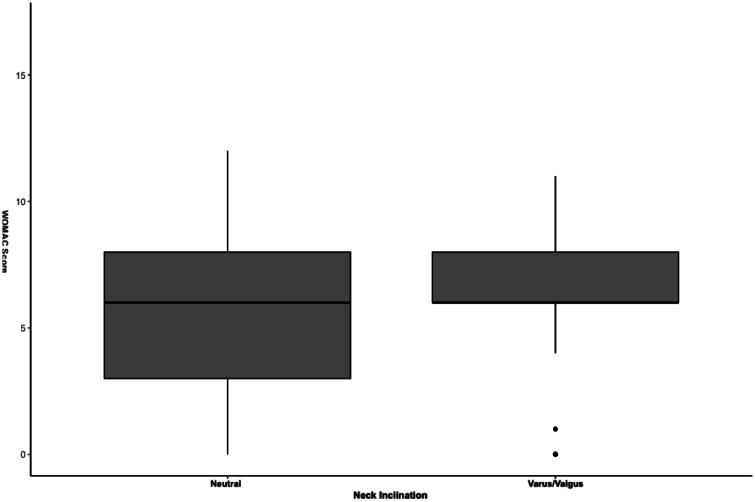
Box plot distribution of WOMAC score values in the 2 subgroups obtained considering the inclination of the neck, *P*-value = .78.

**Figure S10:**
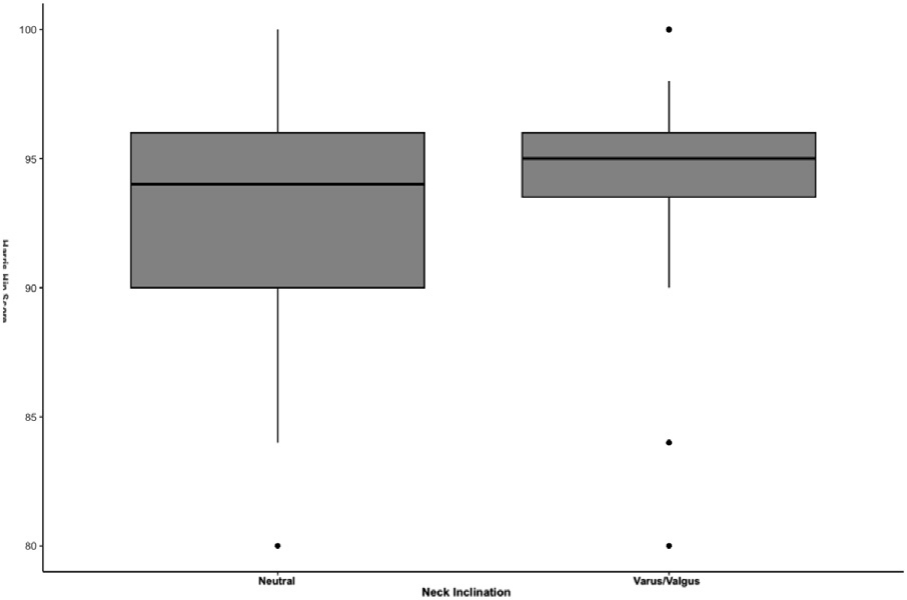
Box plot distribution of Harris hip score values in the 2 subgroups obtained considering the inclination of the neck, *P*-value = .94.

**Figure S11:**
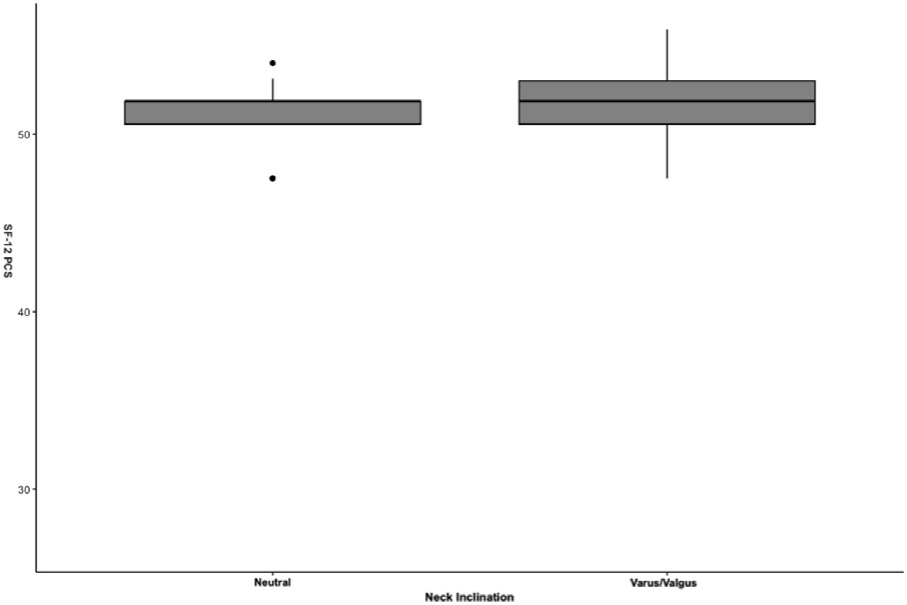
Box plot distribution of SF-12 PCS values in the 2 subgroups obtained considering the inclination of the neck, *P*-value = .49.

**Figure S12:**
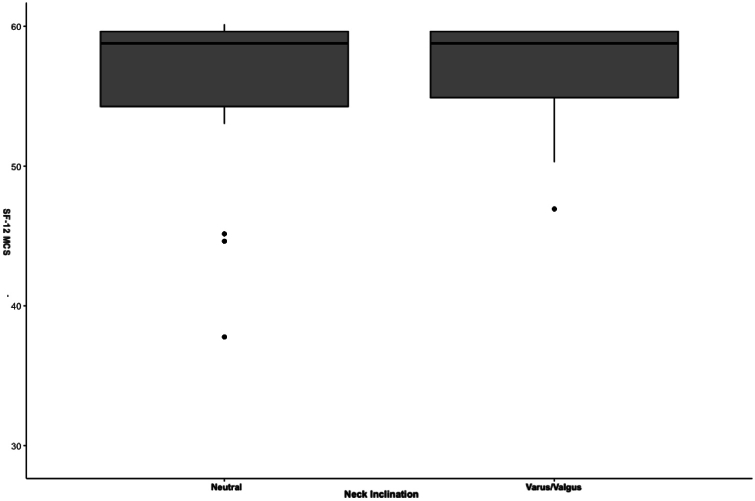
Box plot distribution of SF-12 MCS score values in the 2 subgroups obtained considering the inclination of the neck, *P*-value = .63.

Subdividing the sample based on neck angle yielded the following results: WOMAC score of 6 for neutral neck and 5.81 for varus/valgus neck (*P*-value = .78) (Fig. S9), HHS of 93.56 for neutral neck and 93.75 for varus/valgus neck (*P*-value = .94) (Fig. S10), SF-12 PCS of 49.20 for neutral neck and 50.69 for varus/valgus neck (*P*-value = .49) (Fig. S11), and 55.75 and 56.07 for SF-12 MCS in the neutral and varus/valgus neck groups, respectively (*P*-value = .63) (Fig. S12) (Table 5). As showed here, no differences were recorded between the different groups of neck distribution in terms of clinical outcomes.

**Table 5:** Clinical outcomes in the subgroups obtained with neutral neck and varus/valgus neck.

	Neutral neck	Varus/valgus neck	*P*-value
Harris hip score	**93.56**	**93.75**	.94
WOMAC score	**6.00**	**5.81**	.78
SF-12			
PCS	**49.20**	**50.69**	.49
MCS	**55.75**	**56.07**	.63

## Discussion

The type of short stem implant with neck preservation studied has proven to be a reliable implant, offering excellent results in terms of patient satisfaction and functional recovery. The results of this study demonstrate that the mid-term survival of the implant is 96.7% with a 10-year revision rate of 1.3%, compared to a 4.8% revision rate and a 95.2% survival rate reported in the Australian Orthopaedic Association National Joint Replacement Registry based on 53,976 standard stem implants [14]. Although literature data following in vitro analyses report a higher percentage of micromovements in the neck preservation stem category [27], this study did not report any cases of aseptic loosening at 6 years, and the one case of a high-energy trauma-related periprosthetic fracture showed a stable stem even following intraoperative testing. As a result, fracture fixation only was performed.

Interesting conclusions can be drawn by comparing the clinical results obtained with a healthy population divided by age groups. Indeed, McLean et al. [24] reported a mean HHS of 95.64 in a sample of 627 healthy subjects, compared to the value of 93.65 in this sample. Bellamy and colleagues reported a WOMAC score of 5.82 in a sample of 5492 subjects from the healthy population, compared to the score of 5.92 here presented [25]. Regarding SF-12, Galenkamp et al. reported a mean PCS of 50.3 and MCS of 51.3 in a sample of 3742 subjects from the European healthy population [26], whereas in this study, an SF-12 PCS of 49.88 and an SF-12 MCS of 55.89 were obtained. This suggests that patients undergoing THA surgery, even at a distance of 6 years, continue to experience levels of satisfaction even higher than those of healthy population but with functional results lower than those of healthy population. From the authors perspectives, the small functional loss has no impact on the patient’s perception of their physical and mental status.

Neck's modularity still causes concern due to the implant’s wear and/or breakage, which could lead to disastrous outcomes such as some modular implant designs have done in the past [28,29]. From our experience, we did not observe any early neck wear or premature failures, in line with literature data based on a simulated 20 years of use, thanks in part to the titanium alloy composition of the neck [30,31].

Solarino et al., in a recent study, demonstrate that despite concerns expressed by many surgeons in various countries, the use of modular neck prostheses in THA remains a beneficial treatment for hip osteoarthritis in cases of hip dysplasia or severely deformed femur [14]. The majority of published studies report clinical outcomes after THA with modular neck prostheses comparable to those achieved with monobloc stems. New generations of implants and the use of femoral necks made from advanced titanium alloys reduce the risk of wear and adverse local tissue reactions, which are the most common complications observed in the analyzed studies.

Traina et al. verify that the malfunction of modular necks is associated with factors such as neck offset, stem size (increased risk with larger stems), and body weight [32]. The authors propose that when encountering conditions such as a high BMI, the likelihood of failure substantially rises. In such cases, the application of modular prostheses should be carefully assessed on a case-by-case basis.

Additionally, considering that femoral offset varies between 27 and 57 mm [33], having a modular neck available is valuable as it allows us to properly restore the native femoral offset [34], avoiding the risk of implant dislocation [35], early insert wear [36], and/or unfavorable biomechanical loading of the prosthetic implant [37]. Moreover, in cases where the patient presents atypical femoral morphology, it allows us to implant a correctly sized stem without the risk of intraoperative fracture, femoral mismatch, or thigh pain in the postoperative period, as reported in the literature following the use of standard stems [38,39].

However, we still suggest using a short neck with a neutral version whenever possible. Although not statistically significant and thus requiring further investigation, we found a trend toward better scores using different combinations of neck versions (HHS: neutral neck 94.35 and anteverted neck 91.41, *P*-value = .06). Is it the author’s opinion that a partial collum stem needs less biomechanical adjustments compared to standard stems due to the small amount of head/neck system to restore? Nevertheless, in those cases, the correction that may be needed has to be very precise and subtle, and the modularity here presented allows it.

The main limitation of the study arises from its retrospective nature. Moreover, the study lacks comparison between control group of standard stems and/or control group with the same monobloc short stem; instead, it relies on clinical data reported in the literature. Therefore, the results here presented cannot be generalized. A strength of the study is the relatively long follow-up, which is uncommon in this particular combination of characteristics (short, neck-preserving stem with modular neck).

## Conclusions

These findings support the claim that, in terms of both survival and clinical results, the short stem prosthesis with femoral neck preservation is a viable implant. It guarantees excellent osteointegration and a low rate of complications; it should be taken into consideration for a group of active patients with a good bone stock. Specifically, this implant, combined with the option to use a modular neck, allows the orthopaedic surgeon to best replicate the patient's anatomy by restoring the correct femoral offset while respecting the tension and length of the surrounding soft tissues.

Furthermore, considering that this type of implant is intended for young and active patients, the stem survival should be equal to or even better than that of standard stems. Therefore, the duration of follow-up should be extended in the years to come to validate these promising results.

## Conflicts of interest

The authors declare there are no conflicts of interest.

For full disclosure statements refer to https://doi.org/10.1016/j.artd.2024.101387.

## CRediT authorship contribution statement

**Michele Carnovale:** Writing – review & editing, Writing – original draft, Software, Methodology, Conceptualization. **Daniele De Meo:** Writing – review & editing, Writing – original draft, Project administration, Methodology, Conceptualization. **Giovanni Guarascio:** Writing – review & editing, Writing – original draft, Investigation, Data curation. **Paolo Martini:** Writing – review & editing, Investigation, Formal analysis, Data curation. **Gianluca Cera:** Writing – review & editing, Validation, Resources, Methodology. **Pietro Persiani:** Writing – review & editing, Visualization, Validation, Resources. **Vittorio Candela:** Writing – review & editing, Validation, Funding acquisition, Formal analysis. **Stefano Gumina:** Writing – review & editing, Validation, Supervision, Project administration, Funding acquisition. **Ciro Villani:** Writing – review & editing, Supervision, Resources, Project administration, Conceptualization.

## Appendix A Supplementary Data

Conflict of Interest Statement for All the Authors
