# Supplementary material for: Mid-Term Outcomes of a Short Modular Neck-Preserving Cementless Hip Stem: A Retrospective Study With a 6-Year Minimum Follow-Up
Source: Arthroplast Today. 2024 Apr 27;27:101387. doi: 10.1016/j.artd.2024.101387 (PMC11068503; doi:10.1016/j.artd.2024.101387)
Supplement: Conflict of Interest Statement for All the Authors [file mmc1.pdf]

CONFLICT OF INTEREST STATEMENT

American Association of Hip and Knee Surgeons

(Adopted from the American Academy of Orthopaedic Surgeons disclosure statement)

Manuscript Title: Mid-term Outcomes of a Short Modular Neck-Preserving cementless Hip Stem: A Retrospective Study with a 6-Year Minimum Follow-up.

1. Royalties from a company or supplier: None

2. Speakers bureau/paid presentations for a company or supplier: None

3A. Paid employee for a company or supplier: None

3B. Paid consultant for a company or supplier: None

3C. Unpaid consultants for a company or supplier: None

4. Stock or stock options in a company or supplier: None

5. Research support from a company or supplier as a Principal Investigator: None

6. Other financial or material support from a company or supplier: None

7. Royalties, financial or material support from publishers: None

8. Medical/Orthopaedic publications editorial/governing board: None

9. Board member/committee appointments for a society: None

Author Name	Author Signature	Date
DANIELE DE MEO	[signature]	1/12/2023

CONFLICT OF INTEREST STATEMENT

American Association of Hip and Knee Surgeons

(Adopted from the American Academy of Orthopaedic Surgeons disclosure statement)

Manuscript Title: Mid-term Outcomes of a Short Modular Neck-Preserving cementless Hip Stem: A Retrospective Study with a 6-Year Minimum Follow-up.

1. Royalties from a company or supplier: None

2. Speakers bureau/paid presentations for a company or supplier: None

3A. Paid employee for a company or supplier: None

3B. Paid consultant for a company or supplier: None

3C. Unpaid consultants for a company or supplier: None

4. Stock or stock options in a company or supplier: None

5. Research support from a company or supplier as a Principal Investigator: None

6. Other financial or material support from a company or supplier: None

7. Royalties, financial or material support from publishers: None

8. Medical/Orthopaedic publications editorial/governing board: None

9. Board member/committee appointments for a society: None

Author Name	Author Signature	Date
PiETRO PERSIANI	[signature]	2-12-2023

CONFLICT OF INTEREST STATEMENT

American Association of Hip and Knee Surgeons

(Adopted from the American Academy of Orthopaedic Surgeons disclosure statement)

Manuscript Title: Mid-term Outcomes of a Short Modular Neck-Preserving cementless Hip Stem: A Retrospective Study with a 6-Year Minimum Follow-up.

1. Royalties from a company or supplier: None

2. Speakers bureau/paid presentations for a company or supplier: None

3A. Paid employee for a company or supplier: None

3B. Paid consultant for a company or supplier: None

3C. Unpaid consultants for a company or supplier: None

4. Stock or stock options in a company or supplier: None

5. Research support from a company or supplier as a Principal Investigator: None

6. Other financial or material support from a company or supplier: None

7. Royalties, financial or material support from publishers: None

8. Medical/Orthopaedic publications editorial/governing board: None

9. Board member/committee appointments for a society: None

Author Name	Author Signature	Date
Ciro Villani	[signature]	1/12/2023

CONFLICT OF INTEREST STATEMENT

American Association of Hip and Knee Surgeons

(Adopted from the American Academy of Orthopaedic Surgeons disclosure statement)

Manuscript Title: Mid-term Outcomes of a Short Modular Neck-Preserving cementless Hip Stem: A Retrospective Study with a 6-Year Minimum Follow-up.

1. Royalties from a company or supplier: None

2. Speakers bureau/paid presentations for a company or supplier: None

3A. Paid employee for a company or supplier: None

3B. Paid consultant for a company or supplier: None

3C. Unpaid consultants for a company or supplier: None

4. Stock or stock options in a company or supplier: None

5. Research support from a company or supplier as a Principal Investigator: None

6. Other financial or material support from a company or supplier: None

7. Royalties, financial or material support from publishers: None

8. Medical/Orthopaedic publications editorial/governing board: None

9. Board member/committee appointments for a society: None

Author Name	Author Signature	Date
PAOLO MARTINI	[signature]	2-12-23

CONFLICT OF INTEREST STATEMENT

American Association of Hip and Knee Surgeons

(Adopted from the American Academy of Orthopaedic Surgeons disclosure statement)

Manuscript Title: Mid-term Outcomes of a Short Modular Neck-Preserving cementless Hip Stem: A Retrospective Study with a 6-Year Minimum Follow-up.

1. Royalties from a company or supplier: None

2. Speakers bureau/paid presentations for a company or supplier: None

3A. Paid employee for a company or supplier: None

3B. Paid consultant for a company or supplier: None

3C. Unpaid consultants for a company or supplier: None

4. Stock or stock options in a company or supplier: None

5. Research support from a company or supplier as a Principal Investigator: None

6. Other financial or material support from a company or supplier: None

7. Royalties, financial or material support from publishers: None

8. Medical/Orthopaedic publications editorial/governing board: None

9. Board member/committee appointments for a society: None

Author Name	Author Signature	Date
GIANLUCA CERA		3/12/2023

CONFLICT OF INTEREST STATEMENT

American Association of Hip and Knee Surgeons

(Adopted from the American Academy of Orthopaedic Surgeons disclosure statement)

Manuscript Title: Mid-term Outcomes of a Short Modular Neck-Preserving cementless Hip Stem: A Retrospective Study with a 6-Year Minimum Follow-up.

1. Royalties from a company or supplier: None

2. Speakers bureau/paid presentations for a company or supplier: None

3A. Paid employee for a company or supplier: None

3B. Paid consultant for a company or supplier: None

3C. Unpaid consultants for a company or supplier: None

4. Stock or stock options in a company or supplier: None

5. Research support from a company or supplier as a Principal Investigator: None

6. Other financial or material support from a company or supplier: None

7. Royalties, financial or material support from publishers: None

8. Medical/Orthopaedic publications editorial/governing board: None

9. Board member/committee appointments for a society: None

Author Name	Author Signature	Date
**Giovanni Guarascio**		**03/12/2023**

CONFLICT OF INTEREST STATEMENT

American Association of Hip and Knee Surgeons

(Adopted from the American Academy of Orthopaedic Surgeons disclosure statement)

Manuscript Title: Mid-term Outcomes of a Short Modular Neck-Preserving cementless Hip Stem: A Retrospective Study with a 6-Year Minimum Follow-up.

1. Royalties from a company or supplier: None

2. Speakers bureau/paid presentations for a company or supplier: None

3A. Paid employee for a company or supplier: None

3B. Paid consultant for a company or supplier: None

3C. Unpaid consultants for a company or supplier: None

4. Stock or stock options in a company or supplier: None

5. Research support from a company or supplier as a Principal Investigator: None

6. Other financial or material support from a company or supplier: None

7. Royalties, financial or material support from publishers: None

8. Medical/Orthopaedic publications editorial/governing board: None

9. Board member/committee appointments for a society: None

Author Name	Author Signature	Date
MICHELE CARNOVALE	[signature]	03/12/23

CONFLICT OF INTEREST STATEMENT

American Association of Hip and Knee Surgeons

(Adopted from the American Academy of Orthopaedic Surgeons disclosure statement)

Manuscript Title: Mid-term Outcomes of a Short Modular Neck-Preserving cementless Hip Stem: A Retrospective Study with a 6-Year Minimum Follow-up.

1. Royalties from a company or supplier: None

2. Speakers bureau/paid presentations for a company or supplier: None

3A. Paid employee for a company or supplier: None

3B. Paid consultant for a company or supplier: None

3C. Unpaid consultants for a company or supplier: None

4. Stock or stock options in a company or supplier: None

5. Research support from a company or supplier as a Principal Investigator: None

6. Other financial or material support from a company or supplier: None

7. Royalties, financial or material support from publishers: None

8. Medical/Orthopaedic publications editorial/governing board: None

9. Board member/committee appointments for a society: None

Author Name	Author Signature	Date
VITTORIO CANDELA	[signature]	03/12/23

CONFLICT OF INTEREST STATEMENT

American Association of Hip and Knee Surgeons

(Adopted from the American Academy of Orthopaedic Surgeons disclosure statement)

Manuscript Title: Mid-term Outcomes of a Short Modular Neck-Preserving cementless Hip Stem: A Retrospective Study with a 6-Year Minimum Follow-up.

1. Royalties from a company or supplier: None

2. Speakers bureau/paid presentations for a company or supplier: None

3A. Paid employee for a company or supplier: None

3B. Paid consultant for a company or supplier: None

3C. Unpaid consultants for a company or supplier: None

4. Stock or stock options in a company or supplier: None

5. Research support from a company or supplier as a Principal Investigator: None

6. Other financial or material support from a company or supplier: None

7. Royalties, financial or material support from publishers: None

8. Medical/Orthopaedic publications editorial/governing board: None

9. Board member/committee appointments for a society: None

Author Name	Author Signature	Date
Prof. STEFANO GUMiNA		03/12/23
